# Promising Bioregulators for Higher Water Productivity and Oil Quality of Chia under Deficit Irrigation in Semiarid Regions

**DOI:** 10.3390/plants12030662

**Published:** 2023-02-02

**Authors:** Chowdasandra Byregowda Harisha, Vijaykumar B. Narayanpur, Jagadish Rane, Vasant M. Ganiger, Sugooru M. Prasanna, Yeragenahalli Chandrashekaharappa Vishwanath, Sanjeevraddi G. Reddi, Hanamant M. Halli, Karnar Manjanna Boraiah, Patil Siddanagouda Basavaraj, Eman A. Mahmoud, Ryan Casini, Hosam O. Elansary

**Affiliations:** 1 ICAR–National Institute of Abiotic Stress Management, Baramati, Pune 413115, Maharashtra, India; 2 College of Horticulture, University of Horticultural Sciences, Bagalkot 587104, Karnataka, India; 3 Department of Food Industries, Faculty of Agriculture, Damietta University, Damietta 34511, Egypt; 4 School of Public Health, University of California, 2121 Berkeley Way, Berkeley, CA 94704, USA; 5 Department of Plant Production, College of Food & Agriculture Sciences, King Saud University, P.O. Box 2460, Riyadh 11451, Saudi Arabia

**Keywords:** chia, deficit irrigation, bioregulators, water productivity, fatty acids

## Abstract

Appropriate water management practices are essential for the successful cultivation of chia in water-scarce situations of semiarid regions. This is highly essential when new crops such as chia are introduced for ensuring diversity and water saving. Therefore, field trials (2020–21 and 2021–22) were conducted to understand the impact of deficit irrigation and bioregulators (BRs) on the seed yield, water productivity, and oil quality of chia. The effect of foliar application of BRs such as thiourea (TU; 400 ppm), salicylic acid (SA; 1.0 mM), potassium nitrate (KN; 0.15%), potassium silicate (KS; 100 ppm), kaolin (KO; 5%), and sodium benzoate (SB; 200 ppm) were monitored at different levels of irrigation: 100 (I_100_), 75 (I_75_), 50 (I_50_), and 25 (I_25_) percent of cumulative pan evaporation (CPE). Deficit irrigation at I_25_, I_50_, and I_75_ led to 55.3, 20.1, and 3.3% reductions in seed yield; 42.5, 22.5, and 4.2% in oil yield; and 58.9, 24.5, and 5.7% in omega–3 yield, respectively, relative to I_100_. Bioregulators could reduce the adverse impact of water deficit stress on seed, oil, and omega–3 yield. However, their beneficial effect was more conspicuous under mild water stress (I_75_), as revealed by higher seed yield (4.3–6.9%), oil yield (4.4–7.1%), and omega–3 yield (4.7–8.5%) over control (I_100_ + no BRs). Further, BRs (KN, TU, and SA) maintained oil quality in terms of linolenic acid and polyunsaturated fatty acid contents, even under mild stress (I_75_). Foliar application of KN, TU, and SA could save water to an extent of 36–40%. Therefore, the adverse impact of deficit irrigation on seed, oil, and omega–3 yields of chia could be minimized using BRs such as KN, TU, and SA, which can also contribute to improved water productivity.

## 1. Introduction

Chia (*Salvia hispanica* L.) is an annual short-day plant of the *Lamiaceae* family, having great demand for health benefits in terms of useful nutrients and Omega–3 fatty acids. Though the plant originated in Southern Mexico and Northern Guatemala, it is now widely grown in Columbia, Peru, America, Bolivia, Argentina, Europe, Brazil, and Australia [1,2]. In southern India, the crop is grown in parts of Karnataka, Tamil Nadu, Madhya Pradesh, and Kerala states. This area expansion is attributed to the recently enhanced demand from industries, which harness the potential of chia seeds and oil for medicines and health benefits. Chia is cultivated for seeds and used as food besides its many industrial applications such as stabilizers [3], emulsifiers or binders in the food industry [4], biodegradable films [5], anticorrosive agents, cosmetics, and pharmaceuticals [6]. Chia seeds are a rich source of fixed oil, ranging from 20.3 to 38.6%, and has high linolenic acid content (55%) [7]. In addition, it has high protein content with a highly balanced proportion of essential amino acids [8]. These nutritional properties make an important raw material for developing health products such as omega-3 capsules, seed mucilage, and seed oil [9]. With the current international market for chia amounting to USD 194.1 million as reported in 2021, market insights have projected the possibilities of further expansion at least by a 6.8% cumulative growth rate, as the consumption of this crop is likely to be 80.57 m MT by 2031 (https://www.futuremarketinsights.com/reports/chia-seed-market#:~:text=%5B367%20Pages%20Report%5D%20Future%20Market,period%2C%20from%202021%20to%202031, accessed on 28 September 2022).

Under restricted access to irrigation, the effective use of available water to improve yield and water productivity is highly important in water scarcity scenarios [10,11]. Among possible options, the adoption of appropriate climate-resilient crops with relatively higher productivity under limited resource conditions could serve this purpose [12]. Interestingly, chia is drought-tolerant and can be grown in tropical-to-desert conditions [13]. However, scientific leads on the water requirement of chia and the impact of deficit irrigation on yield, quality, and water productivity are less understood. Therefore, deficit irrigation is considered the key strategy to knowing the stress tolerance and yield potential [14]. It hypothesized that chia crops can be grown in water-scarce situations, since they can extract water from deeper layers of soil under varying degrees of water deficits. Thus, understanding the effect of irrigation water levels on crop performance during the entire cropping period seems essential for planning irrigation strategies. From the limited studies carried out with chia, it is evident that deficit irrigation reduces seed yield and oil yield by 33 and 5%, respectively, when irrigation is restricted to 40% evapotranspiration (ET) [13]. In another study by De Falco et al. (2018) [15], there was an increase in oil content by 7.5% under irrigated conditions compared to the rainfed crop. Deficit irrigation induced an increase in the water use efficiency reported in other crops such as chia [13], nigella [16], wheat [17], and coriander [18] due to the effective conversion of photoassimilates to reproductive yield.

In addition to the selection of suitable crops, several studies have suggested the beneficial effect of exogenous bioregulators (BRs) and nutrient supplements in mitigating the water deficit stress under both fields and controlled conditions [19,20]. These BRs impart stress tolerance in plants through sustained physiological processes, and thereby improve plant growth, nutrient translocation, and source-to-sink transitions [21]. Among several BRs, thiourea (TU) was found to be efficient in the alleviation of drought stress in plants as it facilitated the production of osmolytes, efficient nutrient uptake, and antioxidant mechanisms [22], as reported in maize, wheat, onion, and spices under soil moisture stress [23,24]. Similarly, KNO_3_ is a nutrient supplement that can supply both K and N, which contribute to the efficient regulation of stomata and synthesis of photosynthetic pigments, respectively [25]. Its positive influence on growth has been reported in black gram [26] and sunflower [27]. Salicylic acid (SA) is another well-known stress signal molecule that activates the stress-responsive gene, which regulates enzymes and proteins that influence stomata closure, nutrient uptake, chlorophyll degradation, and photosynthesis [28]. Many studies in wheat, fennel, coriander, and chickpea crops demonstrated the positive effect of SA in protecting against drought-induced oxidative damage [17,29,30,31]. Kaolin is a reflective material that reduces the absorption of ultraviolet and infrared rays, and thus can maintain a cooler canopy and reduce radiation stress. The role of antitranspirants such as kaolin in imparting stress tolerance is demonstrated in wheat [32], nuts [33], and apples [34]. Thus, kaolin is a cheap and alternate option for alleviating water deficit stress in plants. Silicon is also a beneficial nutrient element for plant growth, and can enhance drought stress tolerance by maintaining plant water status, resulting in improved crop yield and quality [35]. The stress-alleviating effects of silicon under moisture deficit have been observed in a wide range of crops, including wheat [36], sunflower [37], soybean [38], and mango [39], suggesting that this beneficial nutrient can be one of the potential bioregulators.

Overall, these reports signify the beneficial role of exogenous application of BRs and nutrient supplements in imparting tolerance against water deficit stress by improving physiological processes and plant–water relations, as well as by induction of the antioxidant defense mechanism [40] and its potentiality in alleviating water stress, as demonstrated in wheat [17] and onion [41]. Despite the suitable soil and climate for chia cultivation in a large part of India, the requisite information on irrigation requirements and best agronomic practices under water scarcity is insufficient. Further, there is a lack of information on the potential benefits of bioregulator applications in chia. Hence, the study was designed to provide insights into the impact of deficit irrigation and bioregulators on seed, oil, and Omega–3 yields of chia, and to assess the potential benefit of bioregulators in improving water productivity in semiarid regions of India.

## 2. Results and Discussion

Crop per drop of water is the trending concept to make agriculture sustainable and profitable for farmers constrained by limited resources, climate uncertainties, and extremes. In this context, persistent efforts are being made to evolve water-saving technologies that include deficit irrigations and bioregulators that can influence the physiology of water relations in plants. Since information on these aspects of potential technologies is missing in the case of chia, the present experiment was designed based on the scientific leads that emerged In this area of crop science to answer questions such as the following: (a) What is the extent of yield losses in chia under severe water deficit conditions?; (b) What is the efficacy of potential BRs reported to be stress alleviators in other crops?; (c) What is the maximum water productivity that could be achieved without yield penalty in chia in the semiarid region of India?; (d) What is the effect of water deficit stress on oil and omega–3 contents with or without BRs under water deficit stress conditions? This section has been oriented to answer these questions based on the data generated in the present investigation and the support that could be derived from the scientific literature.

### 2.1. Climatic Conditions of the Study Location

**Figure 1:**
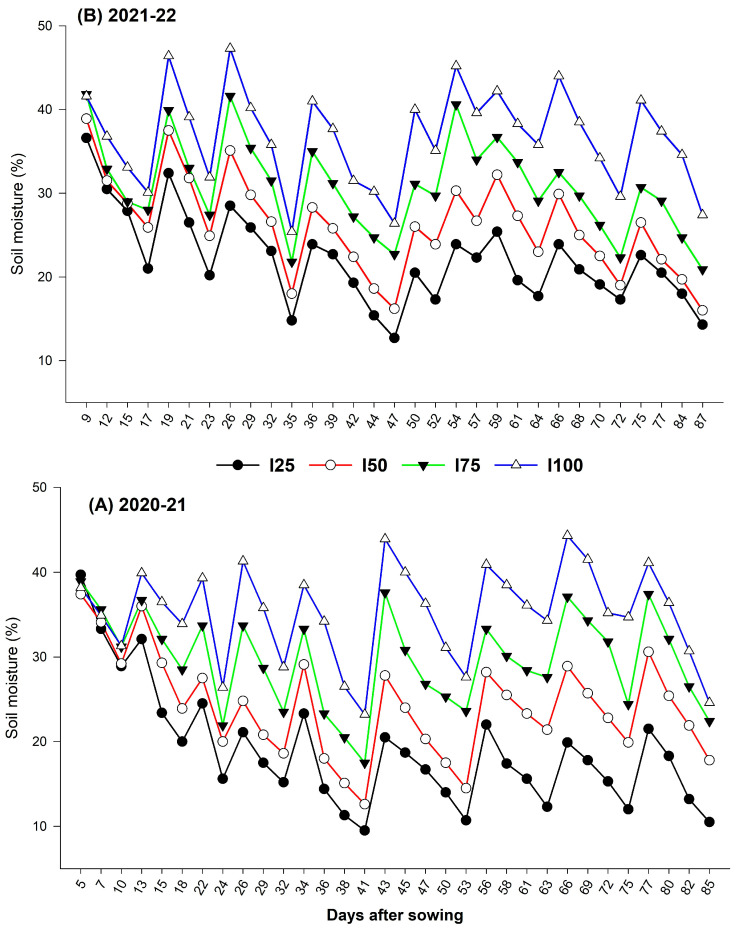
Year–wise soil moisture distribution due to irrigation regimes throughout the chia growth stages.

All growth, yield, and quality parameters were comparatively higher during 2020–21 than in 2021–22. The varied response in seed yield was obviously due to the relatively hot and dry environment during 2020–21 (Table 1). Compared to 2021–22, the max day temperature was higher by 2.9 °C at the flowering stage, and therefore, there was higher CPE (125.3 mm) and lower relative humidity (64.9%) at the seed setting stage during 2020–21. Though the chia was sown on 13 November during both years, an additional 10.6 mm of precipitation and a higher amount of irrigation water during 2020–21 at the flowering stage might have resulted in better growth, seed set, yield, and quality of chia in all the treatments. On average, the soil moisture content ranged between 15–38% during both years (Figure 1). There was a reduction in soil moisture by 50, 34, and 17% due to irrigation levels I_25_, I_50_, and I_75_, respectively, compared to I_100_. An earlier study by Ayerza (1995) [42] and Baginsky et al. (2016) [43] showed that higher yield and oil quality were observed when chia was cultivated under dry climate when temperature ranged from 15 to 29 °C with adequate soil moisture at flowering and seed set. 

### 2.2. Effect of Water Deficit on Seed, Oil, and Omega–3 Yield

The seed, oil, and omega–3 yields of chia were significantly influenced by irrigation regimes and BRs (Table 2). The average seed yield due to irrigation regimes ranged from 323.1 kg ha^–1^ in I_25_ to 723.7 kg ha^–1^ in I_100_ across years. The seed yield was higher during 2020–21, which might be due to the receipt of 10.6 mm rain during the active growth phase at 60 days after sowing, as well as due to the crop being irrigated with a higher amount of water compared to 2021–22. The results showed that the seed yield was reduced by 3.3, 20.1, and 55.3% under I_75_, I_50_, and I_25_, respectively.

Similarly, the oil yield of chia ranged from 101.5 kg ha^–1^ in I_25_ to 238.3 kg ha^–1^ in I_100_ across the years and BR treatments. The reduction in oil yield compared to I_100_ was 4.2, 22.3, and 57.4% under I_75_, I_50,_ and I_25_, respectively. The Omega–3 yield of chia ranged from 52.5 (I_25_) to 127.8 (I_100_) kg ha^–1^, while the reduction in response to water deficit was 6, 25, and 41% under I_75_, I_50,_ and I_25_, respectively. The reduction in seed, oil, and Omega–3 yield of chia under deficit irrigation was primarily due to a reduction in yield determinants such as the number of spikes per plant, spike length, test weight, oil content, harvest index, and omega–3 content in oil (Table 3). As evident from the data, the reduction in the number of spikes, length of spikes, 1000-seed weight, oil content, and harvest index (HI) was 5.1 to 24.5, 5.7 to 30.6, 2.4 to 8.1, and 0.9 to 4.6% in response to I_25_ and I_75_, respectively, relative to I_100_, whereas the deficit irrigation of I_75_ resulted in the highest HI (20.2), followed by I_50_, I_100_, and I_25_. These results indicate the negative impact of deficit irrigation on chia yield in proportion to the reduction in applied water. In previous studies, the reduction in seed yield in chia was attributed to the reduction in the density of seeds per spike under deficit irrigation [13]. Results from our study align with reports on the impact of deficit irrigation on camelina [44], canola [45], and safflower yield [46].

Further, our results hint that the reduction in seed yield was the primary reason for the decline in oil and omega–3 yield when the crop was subjected to water deficit (Table 2). The decrease in oil yield in chia may be attributed to the reduction in the oil content of seeds (Table 3). These results are in tune with previous investigations in mustard [47], camelina [48], nigella [49], sunflower, safflower, and sesame [50]. The substantial reduction in the test weight of chia seeds observed in our study suggests that the reduced size of the endosperm might have resulted in a decrease in oil content in the seed. This derives support from a previous report on soybean crop [51].

### 2.3. Effect of Bioregulators on Seed, Oil, and Omega–3 Yield

Foliar application of BRs effectively improved seed, oil, and omega–3 yield of chia irrespective of years and irrigation levels. The seed yield of chia was highest in KN-sprayed plants 610.2 kg ha^−1^. The improvement in seed yield due to KO, SA, PS, SB, TU, and KN was 1.8, 9.1, 4.7, 5.4, 9.7, and 11.3%, respectively. Among all the BRs, KN (11.3% of control) and TU (9.7% of control) outperformed others in improving the seed yield (Table 2). The improvement in seed yield was attributed to the higher number of spikes, spike length, and 1000-seed weight due to BRs (Table 3 and Table S1). Foliar application of KN resulted in a higher number of spikes (24.28), spike length (19.68 cm), and 1000-seed weight (1.19 g), at par with TU and SA. The harvest index found to be highest in the KO and control plants may be due to lower vegetative and higher seed biomass. The beneficial effect of KN and TU in water regulations and photosynthesis improved the overall plant physiology and yield attributes under deficit irrigation. Similar improvement in yield attributes due to the positive effect of KN in sunflower [52] and TU in fenugreek [24] and wheat [17] is well documented. The irrigation × BRs interaction effect revealed that the advantage of BRs can be realized under a certain level of deficit irrigation. For instance, the impact of all the BRs except kaolin was almost similar under sufficient irrigation (I_100_). However, KN and TU exhibited a significantly high impact in terms of improved seed yield in I_75_ (5 to 7%) compared to the control (I_100_ without BRs). Further reduction in irrigation water up to I_50_ (no BRs) reduced the yield up to 21.6% relative to control (I_100_ without BRs). However, with the application of BRs such as KN and TU, the yield loss could reduce to 10.5–11.5% compared to the control (I_100_ without BRs) (Table 2).

The oil yield due to various BRs varied between 178.8–198.7 kg ha^−1^, whereas seeds from control plants resulted in the lowest oil yield (171.7 kg ha^−1^). Among all BRs, a higher seed yield was obtained from KN-sprayed plants (198.7 kg ha^−1^) followed by TU (196.2 kg ha^−1^). Similarly, the omega-3 yield was higher in seeds obtained from KN-sprayed plants (106.5 kg ha^−1^), followed by TU (105.0 kg ha^−1^). The improvement in oil and omega–3 yield due to BRs was 4–13.9% and 2–8.5% over the control (no BRs). This improvement was because of higher seed yield; oil content of seeds and omega–3 in oil due to the application of BRs. Application of TU and KN resulted in higher oil content of 32.49 and 32.43%, respectively, which enhanced the oil yield of chia under these treatments. Further, BRs, viz. KN, TU, and SA, at mild deficit irrigation (I_75_) improved oil and omega–3 yield by 6 to 7% and 4.7 to 8.5%, respectively, compared to I_100_ without BRs (Table 2). However, the benefit of BRs in improving oil and omega–3 yield was not so conspicuous in moderate (I_50_) and severe (I_25_) water deficits. The beneficial effect of KN observed in our experiment derives support from earlier studies, which revealed enhanced tolerance to deficit irrigation due to KN in soybean [53]. Similarly, TU effectively improved the seed and oil yield of Indian mustard under water stress conditions [54]. It is important to note that TU contains a substantial proportion of N (36%) and sulfur (42%), which can also contribute to plant growth and development as essential nutrients [55]. Thus, our study supplements the earlier reports on KN and TU as stress alleviators and yield enhancers in crop production under soil moisture deficit. The benefit of these BRs could be realized from the improvement in plant growth driven by key physiological and biochemical processes.

### 2.4. Effect of Deficit Irrigation and BRs on Growth and Physiology of Chia

**Figure 2:**
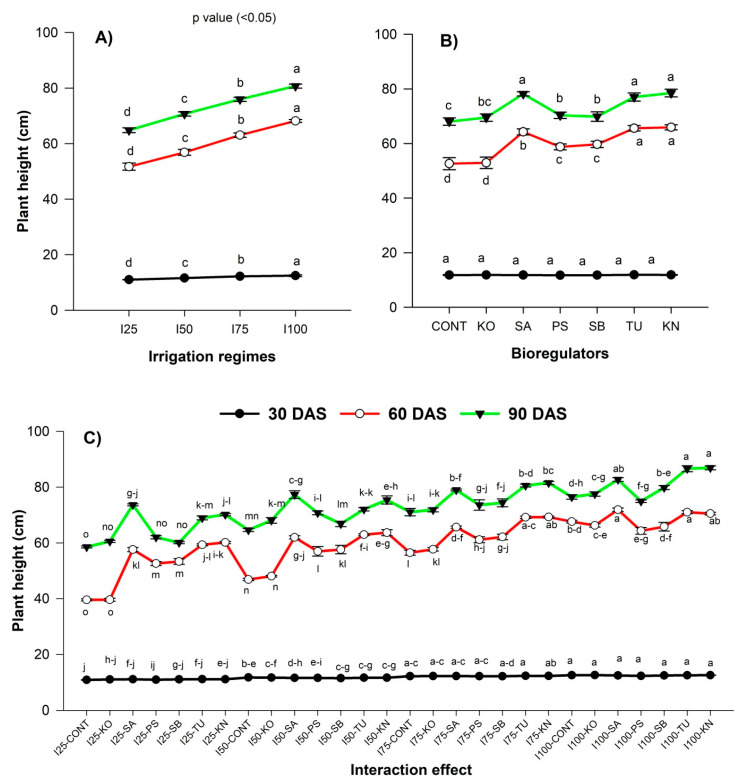
Effect of irrigation level (**A**), BR (**B**), and interaction effect (**C**) on plant height of chia at various growth stages. KO, SA, PS, SB, TU, KN, and no BR denote kaolin, salicylic acid, potassium silicate, sodium benzoate, thiourea, potassium nitrate, and control, respectively, while BRs and CPE denote bioregulators and cumulative pan evaporation, respectively.

**Figure 3:**
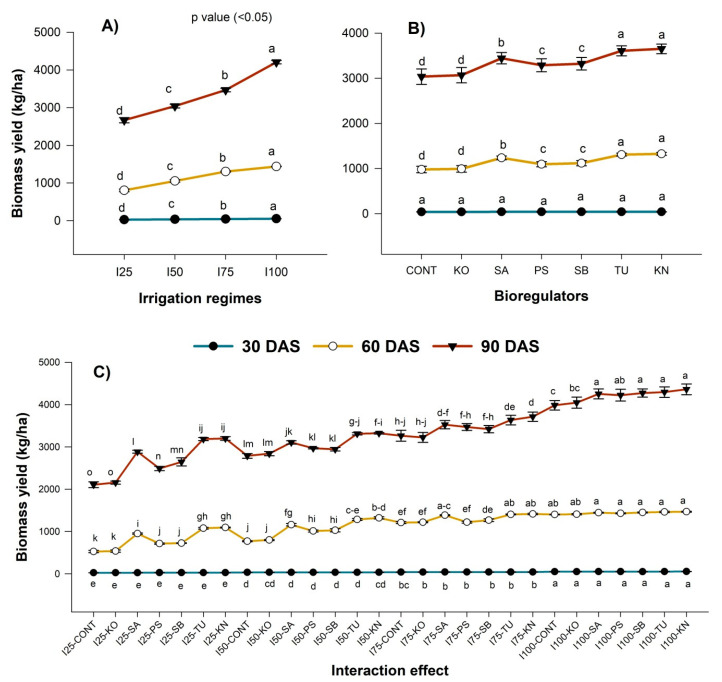
Effect of irrigation levels (**A**) and BRs (**B**) and interaction effect (**C**) on biomass production of chia at various growth stages. KO, SA, PS, SB, TU, KN, and no BR denote kaolin, salicylic acid, potassium silicate, sodium benzoate, thiourea, potassium nitrate, and control, respectively, while BRs and CPE denote bioregulators and cumulative pan evaporation, respectively.

Plant height and biomass production of chia responded significantly to irrigation and BR application at different growth stages. At 30 DAS, the deficit irrigation (I_75_–I_25)_ significantly reduced plant height by 2 to 11.5% (Figure 2A) and biomass by 19.5 to 47.1% (Figure 3A) compared to I_100_. Similarly, the reduction in plant height and biomass of chia was more conspicuous at 60 and 90 DAS. The plant height was lowest in I_25,_ both at 60 (51.7 cm) and 90 DAS (64.84 cm). From the phenology of chia, it was observed that the growth of plants was slower until 30 DAS, and 69% of the growth was achieved between 30–60 DAS. The biomass accumulation also showed that a similar trend was that of plant height. The biomass accumulation ranged between 28.3 and 53.5 kg ha^−1^ during 30 DAS, whereas it was 805.0–1438.7 kg ha^−1^ at 60 DAS. At 90 DAS, the I_25_ resulted in the lowest biomass (2668.0 kg ha^−1^). A similar trend of reduction in plant height and biomass accumulation in chia was reported under deficit irrigation in previous studies [13,56]. The reduction in the growth of plants under deficit irrigation was attributed to the decrease in water and nutrient uptake due to the closing of stomata, poor transpiration, and lower turgor pressure, which reduced photosynthesis and ultimately cell expansion, leading to poor plant growth and dry matter accumulation [57].

As evident from our study, the benefit of BRs in increasing plant height (2–14%) and biomass (1–26%) was conspicuous at 60 DAS (Figure 2B and Figure 3B) compared to without BRs. Among them, KN and TU showed higher benefits in improving the plant growth of chia. Applying KN could improve plant height by 13.2% and biomass by 16.8% at 90 DAS compared to the control. Similarly, the improvement in plant height and biomass was 11.6% and 15.8% with the application of TU compared to the control (Figure 2C and Figure 3C). Such effects of KN and TU may be due to the presence of nitrogen (KN, TU) and potassium (KN), which are the essential nutrients for plants [58]. KN-induced enhancement of plant height and biomass under mild water deficit stress was also reported in groundnut [59]. Similarly, TU supplementation in sunflower improved photosynthesis rate and plant–water relations [60].

**Figure 4:**
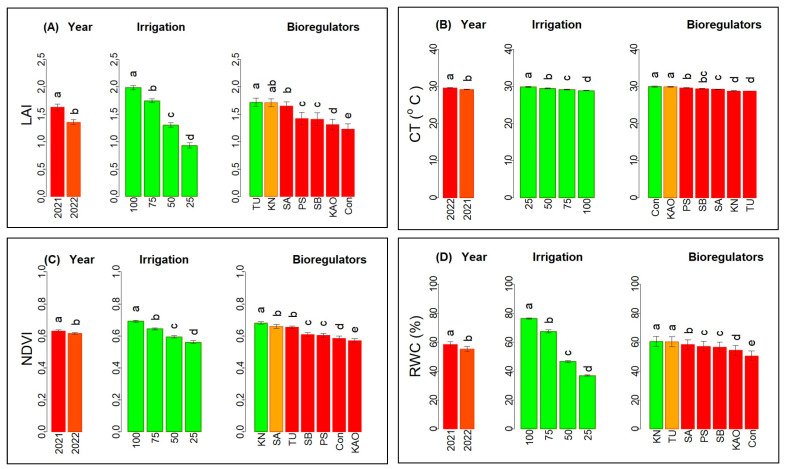
Effect of irrigation and bioregulators on growth and physiology of chia: (**A**) leaf area index (LAI), (**B**) canopy temperature (CT), (**C**) normalized difference vegetation index (NDVI), and (**D**) relative water content (RWC).

The reduction In LAI was proportionate to the reduction in applied water, and it was reduced by 50% at severe stress (I_25_) compared to I_100_ (Figure 4A). This impact of moderate water stress could be alleviated significantly by the foliar spray of KN. The reduction in soil moisture during the study was reflected by a decrease in RWC (67.39 to 37.01%), NDVI (0.64 to 0.56) and increased canopy temperature (29.2 to 29.9 °C) of chia due to deficit irrigation (I_75_–I_25_) compared to I_100_ as shown in Figure 4B–D, respectively. These findings corroborate earlier studies that reported a reduction in RWC in eggplant [61] and maize [62], and NDVI in cluster bean [63], due to reduced water uptake under deficit water stress. As opined by Taghvaeian et al. (2014) [64], the CT can be used as an indicator of moisture deficit stress, since a reduction in plant available water leads to lower transpiration, and consequently, an increase in the CT in sunflower. In the present investigation, the elevated CT (Figure 4B) coincided with the low RWC (Figure 4D) in chia leaves under deficit irrigation (I_25_–I_75_). This adverse impact of deficit irrigation can be overcome by the use of BRs. The beneficial effect of KN could be realized from an improvement in RWC by 16% and NDVI by 14% compared to the control. Consequently, there was a reduction in the CT to the extent of 1.1 °C over plants in control (no BRs). The presence of K^+^ and N in KN helps to maintain leaf water status and stomata regulation, resulting in higher RWC and cooler canopy. Thus, these BRs were essential in modulating canopy temperature and leaf water status under water deficits [23]. The positive effect of KN in osmatic adjustment and maintaining higher RWC under moisture stress tolerance was observed in cotton [65]. Thus, our study suggests that the reduction in plant growth due to deficit irrigation was the primary cause of yield reduction in chia, and that the adverse impact could be reduced by foliar application of KN and TU.

### 2.5. Fatty Acid Composition

Chia oil contains predominantly five fatty acids, namely linolenic acid (18:3), linoleic acid (18:2), oleic acid (18:1), stearic acid (18:0), and palmitic acid (16:0), determined as FAME, as previously observed in other reports of chia [15]. However, the quality of chia is determined by the proportion of nutritionally rich proximate factors such as linolenic (omega–6) and linoleic acid (omega–6) fatty acids in the oil. There was a reduction in the linolenic acid content due to deficit irrigation of I_25_ (51.72%), I_50_ (52.24), and I_75_ (52.77%) compared to I_100_ (53.62%). The linolenic acid reduction was 1.5 to 3.5% due to deficit irrigation (I_75_–I_25_) over I_100_. The linoleic acid content also showed a similar trend as that of linolenic acid. The lowest linoleic acid was observed in I_25_ (22.90%) and the highest was in I_100_ (24.36%). The reduction in linoleic acid was to the extent of 1.5 to 5.9% because of deficit irrigation (I_75_–I_25_) over I_100_. Similar reasons were reported for reduced linolenic acid content in response to deficit irrigation in chia [15] and sunflower [66]. Contrarily, Herman et al. (2016) [13] reported no significant effect of deficit irrigation on omega–3 content in chia due to a cooler climate existing in high altitudes. However, in our study, the dry and warm climate might also be responsible for variations in fatty acids under stress conditions.

Contrarily, there was an increase in stearic acid by 6.2–24.6% and palmitic acid by 5.9–9.2%, across deficit irrigations (I_25_–I_75_) compared to full irrigation (I_100_) (Table 4). The present study also revealed an increase in oleic acid content in oil due to water stress (I_75_–I_25_) (Table 4). The oleic acid content was higher in I_25_ (9.16%) followed by I_50_ (8.88%). The lowest oleic acid was obtained from I_100_ (8.29%). The reduction in oleic acid due to deficit irrigation was because of the comparative reduction in linolenic acid and linoleic acid. These results corroborate with observations recorded in sunflower under drought stress [67]. The decrease in long-chain fatty acids such as linoleic and linolenic acid could be due to poor translocation of sugars such as glucose, fructose, and sucrose from stem to seeds under drought stress, as observed in soybean [68]. This reduction in unsaturated fats under deficit irrigation contributes to cell membrane stability under stress conditions [69,70]. The ability to regulate membrane fluidity by changing the levels of unsaturated fatty acids is a characteristic of stress-tolerant plants mainly mediated by activities regulated by fatty acid desaturases. This might be one of the reasons for the increased stearic and palmitic acid, and contrarily the reduction in linoleic and linolenic acid. In addition, the reduction in linolenic and linoleic acid was attributed to a possible thermal effect of deficit irrigation on the activity of ‘fatty acid desaturase’ involved in the breakdown of unsaturated fatty acid, as observed in soybean [68] and in safflower [69]. In our study, there was a substantial increase in the canopy temperature of plants in response to deficit irrigation. These results derive support from earlier reports on the adverse impact of elevated temperature on the composition of safflower oil [71] and sunflower [72]. Another possibility is that higher reactive oxygen species production might increase lipid peroxidation, leading to the conversion of unsaturated fats to saturated fats [73].

Our study revealed the beneficial effects of BRs in improving the quality of chia oil by increasing the linoleic and linolenic acids (Table 4). Due to BR application, there was an increase in linolenic acid ranging between 51.89–53.37%. Among BRs, KN was found to be significant in improving the linolenic acid (53.37%) in oil, followed by TU (53.30%) and SA (52.80%). The improvement in linolenic acid due to KN, TU, and SA was 2.7, 2.6, and 1.7% higher than the control, respectively (without BRs) (Table 4). Foliar application of KN resulted in lower stearic acid (4.86) and oleic acid (8.52%) in chia oil, which was 15.6 and 6.4% lower compared to the control, respectively. At the same time, TU resulted in lower palmitic acid (9.17%), which was at par with KN.

Interaction of deficit irrigation and BRs showed that even with a 50% reduction in applied water (I_50_), KN and TU could maintain 52.25–52.95% linolenic acid in chia oil, which was comparable to I_100_ without BRs (Table S2). This indicates the benefit of KN and TU in maintaining the quality of chia oil in terms of high linolenic acid content under moisture deficit stress. The presence of essential nutrients such as N and K (in KN) and S (in TU) might have contributed to the relatively better oil quality of chia, as observed even with sufficient irrigation. Nevertheless, the role of KN and TU in imparting stress tolerance and higher lipid biosynthesis can be the reason for the retention of the oil quality under water stress conditions. This observation aligns with reports on an improvement of fatty acids such as oleic, linoleic, and linolenic acid in safflower [69], camelina oil [74], and soybean [75] when plants were applied with N, S, and K containing nutrient formulations.

### 2.6. Water Productivity for Seed

**Figure 5:**
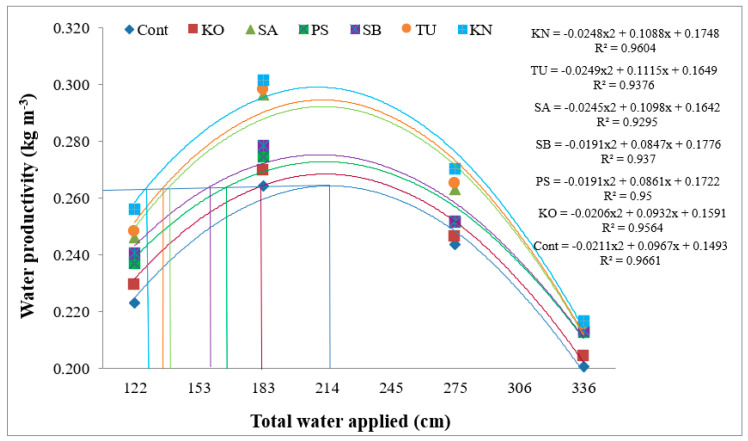
Water productivity functions for chia seed as influenced by bioregulators, viz. cont, KO, PS, SB, SA, TU, and KN. KO—kaolin; KN—potassium nitrate; PS—potassium silicate; SA—salicylic acid; TU—thiourea; SB—sodium benzoate.

Enhanced water productivity (WP) and crop yield are essential for sustainable and climate-resilient agriculture. In this regard, the benefit of BRs has to be evaluated both in terms of the seed yield and WP of chia. The results showed that irrigation levels significantly affected the WP (Figure 5). The range of WP was 0.211 to 0.283 kg m^–3^ across the irrigation regimes. The higher WP for seeds was obtained in I_50_ (0.283 kg m^–3^), followed by I_75_ (0.256 kg m^–3^) and I_25_ (0.243 kg m^–3^), and was lowest in I_100_ (0.283 kg m^–3^). Deficit irrigation improved the WP to the extent of 12.8, 25.4, and 17.5% at severe (I_25_), moderate (I_50_), and mild (I_75_) stress conditions, respectively, compared to no stress (I_100_). Lower WP in nonstress conditions (I_100_) was due to a greater proportion of unutilized biomass in vegetative parts. An increase in WP in the deficit moisture may be attributed to increasing net assimilation or decreasing transpiration, leading to a balanced proportion of seed and vegetative biomass [76]. Further, our study draws support from reports of Herman et al. (2016) [13] in chia, stating that the increase in WP was almost 18% for seed, 50% for oil, and 33.3% for omega–3 under deficit moisture (40% ET). A similar observation of improved WP under deficit irrigation was made in coriander [18], fenugreek [77], and wheat [17]. There was a beneficial effect of BRs on WP, especially under water stress conditions (Figure 5). The WP due to BRs ranged between 0.232–0.261 kg m^–3^. Among BRs, KN was more beneficial than TU and SA. The increment in WP due to KN was 11.1% over control. Further, the effects of TU and SA were also found to be highly significant compared with the control. These results agree with reports that KN and TU were most effective in improving WP due to the presence of K^+^ in KNO_3,_ which maintains the turgidity of guard cells and regulates stomatal mechanisms to reduce transpiration losses of water. This could have significantly improved WP under moderate stress conditions [17,78].

Second-degree polynomial equations could effectively (R^2^ = 0.92–0.96) explain the association between WP and total applied water (Figure 5). WP responses revealed the advantage of BRs over the control. Further, the analysis revealed that the maximum WP for seed achieved without BRs was 0.258 kg m^–3^ at 224 mm of applied water. An equivalent WP for seed could be obtained by KN with only 135 mm of total applied water, suggesting that saving of 40% of irrigation water is possible. Similarly, the equivalent WP for seed could be obtained with BRs at 144, 147, 166, 175, and 192 mm water for TU, SA, SB, PS, and KO, respectively, indicating water saving ranging from 14.3–39.9% with exogenous foliar application of BRs.

### 2.7. Interrelationship between Traits as Influenced by Bioregulators

**Figure 6:**
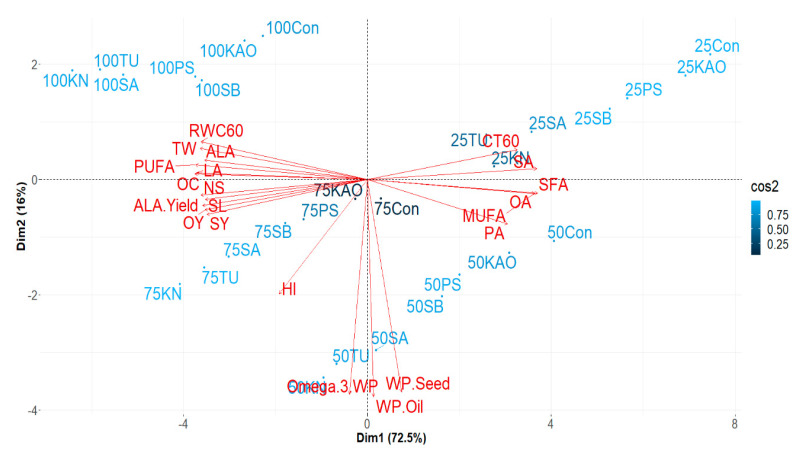
PCA biplot showing interrelation between traits such as seed yield (SY), oil content (OC), oil yield (OY), test weight (TW), number of spikes (NS), spike length (SL), linoleic acid yield (ALA), relative water content (RWC), canopy temperature (CT), polyunsaturated fatty acids (PUFA). Saturated fatty acid (SFA), oleic acid (OA), palmitic acid (PA), stearic acid (SA), linoleic acid (LA), linolenic acid (ALA), water productivity (WP) grown under irrigation regimes (100, 75, 50, and 25% CPE) with various bioregulators. Variables represented by vectors (red markings) and irrigations in loadings (blue markings).

Multivariate analysis was carried out to capture and explain the variability and association among the different traits and bioregulator effects. PC1 and PC2 together accounted for 88.5% of the total variability in the dataset (Figure 6). The principal component analysis could classify the irrigation levels as expected. Among the different parameters considered for analysis, contributions of WP−seed, WP−oil, and WP−omega−3 to total variability were relatively higher than other parameters. KN, TU, and SA were associated with high values of WP−seed, WP−oil, and WP−omega−3, particularly I_50_, suggesting that the benefit of these bioregulators can be realized even with 50% less water.

Under mild or absence of water stress, seed and oil yield were closely associated with yield attributes. HI was also associated with WP−seed, WP−oil, and WP−omega−3, as revealed by the narrow angle between corresponding vectors. This observation aligns with the strong and positive association between oil yield and HI in chia reported by Herman et al. (2016) [13]. Under severe water stress conditions (I_25_), our data revealed that the canopy temperature was positively associated with the accumulation of saturated fatty acids such as palmitic acid, stearic acid, and oleic acid, suggesting that a hotter canopy resulted in the deterioration of oil quality in terms of higher saturated fatty acids (PA, SA, and OA). A possible association between high temperature and fatty acid deterioration was also reported by Oliva et al. (2006) [72] in soybean crop.

## 3. Materials and Methods

### 3.1. Soil and Weather Conditions of the Location

The field experiment was conducted for two consecutive years (2020–21 and 2021–22) at ICAR–National Institute of Abiotic Stress Management, Baramati, Maharashtra, India. The study location is situated at latitude (18°09′30.62″ N), longitude (74°30′03.08″ E), and an altitude of 570 m above mean sea level (MSL). The location is categorized as the hot and semiarid climate of the Deccan Plateau region under agroecological region 6 and water scarcity zone of Maharashtra [79]. The location receives an average annual rainfall of 560 mm, and a substantial proportion is distributed from June to October. The mean yearly United States Weather Bureau (USWB) open pan evaporation of the area is three-fold higher than the mean rainfall (1965 mm). The average maximum temperature during the chia-cropping period ranged between 29.5–30.7 °C, with cumulative evaporation of 475.8–543.1 mm. The detailed weather parameters recorded during cropping seasons (2020–22) are given in Table 1. The soil texture of the experiment site was medium black with sand (11.9%), silt (18.8%), and clay (69.2%), according to Rajagopal et al. (2018) [80]. The chemical properties of the soil were pH (8.19), EC (0.24 dS m^–1^), medium organic carbon (6.5 g kg^–1^), medium in available N (175.0 kg ha^–1^), low in P_2_O_5_ (7.9 kg ha^–1^), and medium in K_2_O (180.0 kg ha^–1^).

### 3.2. Experimental Details and Crop Management

The experiment was laid out in a split-plot design with four irrigation regimes (25, 50, 75, and 100% CPE) in main plots and six bioregulators (BRs: kaolin, salicylic acid, sodium benzoate, potassium silicate, thiourea, potassium nitrate) and control (water spray with no BR) in subplots. Each treatment was replicated three times. The land was prepared for sowing by one deep ploughing followed by harrowing. The white-colored chia seeds were obtained from the University of Horticultural Sciences, Bagalkot, Karnataka, and were sown on 13 November 2020 and 2021. Sowing was carried out at the rate of 2.5 kg ha^–1^ on the flatbeds (3 m × 3 m) by manual dibbling at the spacing of 60 × 30 cm. A buffer area of 1 m was maintained between each block to minimize the lateral movement of soil moisture and nutrients. The nutrients, N:P_2_O_5_:K_2_O at 90:60:75 kg ha^–1^, respectively, were applied through fertilizers [CO(NH_2_)_2_], [Ca (H_2_PO_4_)_2_] and KCl, respectively, whereas 100% (P_2_O_5_ and K_2_O) and 50% of N was applied as a basal dose, and the remaining N was applied at 30 days after sowing (DAS). The weeds were removed manually at 30 and 60 DAS.

### 3.3. Irrigation and Bioregulators Application

Irrigation was delivered through a surface drip irrigation system (Jain Irrigation Systems Ltd., Jalgaon, India) made of 16 mm laterals with inline emitters having a discharge rate of 4 L h^–1^. In each plot, five laterals were placed to have 50 emitters and the irrigation system operated at a pressure of 1.3 kg cm^–2^. The plots were irrigated based on the climatological approach, i.e., the cumulative USWB Class A open pan evaporation (CPE), according to treatments in main plots (I_25_, I_50_, I_75_, and I_100_). Before imposing water deficit, all the plots received two common irrigations (25 mm each) through the drip irrigation method immediately after sowing and a week after sowing for uniform germination and establishment of seedlings. Details of the amount of water applied, the number of irrigations at different crop stages, and the amount of rainfall received during the cropping season are presented in Table 5. The irrigation time to supply the required amount of irrigation water was calculated using the following formula:

(1) $$\mathrm{Irrigation}\;\mathrm{time}\;\left(\min\right)=\frac{\mathrm{Area}\;\left(m^{2}\right)\times \mathrm{CPE}\;\left(\mathrm{mm}\right)\times 60\times \mathrm{Irrigation}\;\mathrm{efficiency}\;\left(\%\right)}{\mathrm{Number}\;\mathrm{of}\;\mathrm{emmitters}\times \mathrm{emitter}\;\mathrm{discharge}\;\left(l/h\right)}$$

Six BRs—thiourea (TU @ 400 ppm), salicylic acid (SA @ 1.0 mM), potassium nitrate (KN @ 0.15%), potassium silicate (PS @ 100 ppm), kaolin (KO @ 5%), sodium benzoate (SB @ 200 ppm)—with control were applied as foliar spray at 30 DAS (seedling), 45 DAS (flower initiation), and 60 DAS (50% flowering). The spray volume of 0.5 L at 30 DAS and 1.0 L per plot (9 m^2^) at 45 and 60 DAS was applied on the following day of irrigation (11.00–13.30 h) with the mentioned concentration of BRs. The control plots without any BRs were sprayed with water equitant to spray volume.

### 3.4. Growth and Physiological Parameters

Chia growth attributes such as plant height and biomass production were recorded at 30, 60, and 90 DAS separately in two years of study. The leaf area of five randomly selected plants in each plot was considered to compute the leaf area index (LAI) at 60 DAS. A leaf area meter (LI–3100 leaf area meter, LI–COR. Inc., Lincoln, NE, USA) was used to ensure precise measurement. In addition, physiological indicators such as normalized difference vegetation index (NDVI) were recorded plotwise at 60 DAS using a handheld Green Seeker Sensor (Trimble, SPL.Tech. Pvt. Ltd., Delhi, India). The canopy temperature (CT) was measured from five randomly selected plants using a handheld infrared thermometer (Agri–therm III™, 6210 L, Everest Interscience Inc., Chino Hills, CA, USA) before noon time at 60 DAS [81]. Relative water content (RWC) of five randomly selected chia leaves from each treatment was determined using the method suggested by Bandyopadhyay et al. [82]. For this second top, fully opened leaves of the chia plant were collected at 12.00 h at 60 DAS. The RWC was calculated using the following formula:

(2) $$\mathrm{RWC}\;\left(\%\right)=\frac{\left(\mathrm{FW}-\mathrm{DW}\right)}{\left(\mathrm{TW}-\mathrm{DW}\right)}\times 100$$

where ‘FW’ is fresh weight, ‘DW’ is dry weight, and ‘TW’ is turbid weight of chia leaves.

### 3.5. Yield and Yield Attributes of Chia

Yield determinants such as the number of spikes per plant, spike length, and 1000-seed weight were recorded at the time of harvest from the selected plants. Then, the seed yield of each plot was recorded after manual threshing and expressed in kg ha^–1^. Later, the harvest index was calculated using the seed yield and biological yield of chia. Similarly, oil and Omega–3 yields of chia were computed using the seed oil content, Omega–3 content, and seed yield, and expressed in kg ha^–1^.

(3) $$\mathrm{HI}\;\left(\%\right)=\frac{\mathrm{Seed}\;\mathrm{yield}\;\left(\mathrm{kg}/\mathrm{ha}\right)}{\mathrm{Biomass}\;\mathrm{yield}\;\left(\mathrm{kg}/\mathrm{ha}\right)}$$

(4) $$\mathrm{Oil}\;\mathrm{yield}\;\left(\mathrm{kg}/\mathrm{ha}\right)=\frac{\mathrm{oil}\;\mathrm{content}\;\left(\%\right)\times \;\mathrm{seed}\;\mathrm{yield}\;\left(\mathrm{kg}/\mathrm{ha}\right)}{100}$$

(5) $$\mathrm{Omega}–3\;\mathrm{yield}\;\left(\mathrm{kg}/\mathrm{ha}\right)=\frac{\mathrm{Omega}–3\;\mathrm{content}\;\left(\%\right)\times \;\mathrm{seed}\;\mathrm{yield}\;\left(\mathrm{kg}/\mathrm{ha}\right)}{100}$$

### 3.6. Water Productivity (WP)

WP of chia was computed by considering the mean yield during both years and the total water supplied [83].

(6) $$\mathrm{WP}\;\mathrm{seed}\;\left({\mathrm{kg}\;m}^{-3}\right)=\frac{\mathrm{Seed}\;\mathrm{yield}\;\left(\mathrm{kg}/\mathrm{ha}\right)}{\mathrm{Total}\;\mathrm{water}\;\mathrm{supplied}\;\left(\mathrm{mm}\right)}\times 10$$

### 3.7. Extraction of Chia Seed Oil

The total oil content (*w*/*w* basis) in chia seeds was extracted by grinding 10 g of clean and dry seeds into the thimble and extracted with 150 mL of n–hexane for 3 h in Soxhlet apparatus following AOCS Method Ba 3–38 [84]. The n–hexane was evaporated and oil weight was recorded to assess the oil content in seed samples. Further, the oil samples were stored at 4 °C for further analysis of fatty acid composition by preparing fatty acid methyl esters. The oil content was expressed as percent of seed weight.

### 3.8. Fatty Acid Methyl Esters (FAME) Analysis

Total fatty acids and fatty acid composition in chia seed oil were determined by preparing FAME according to AOCS (2017) [85] method Ce 2–66. For this, 100 ± 1 mg of oil samples were weighed in reaction vials and 1 mL of BF3–Methanol was added to the mixture. The reaction mixture was heated in sealed vials in water bath at a temperature of 60 °C for 30 min. To the cooled mixture, 3 mL of n–hexane was added to recover the methyl esters to the organic phase, then 1 mL of saturated NaCl solution was added. The aqueous and upper hexane layer was separated to clean vials and anhydrous sodium sulfate was added to remove any moisture in the sample. The hexane was allowed to evaporate and then the residue was stored in −20 °C until further analysis. The FAMEs were diluted in 1 mL of hexane before estimation. Diluted FAME was separated on a GC–MS (Shimadzu QP2020 Kyoto, Japan) equipped with an SH–Rtx–Wax column (30 m × 0.325 mm × 0.25 μm); a sample of 1 μL was used in split mode (10:1) with an autosampler. Helium was the carrier gas at a flow rate of 2.02 mL min^–1^. The column temperature was programmed from 50 to 240 °C with an equilibrium time of 2.5 min, held for 35 min. Injector temperatures were set at 240 °C. The fatty acids were identified by comparing their retention indices, and their identification was confirmed by matching their mass spectra in the NIST–MS library. Each fatty acid content was expressed in percentage based on the retention area in the chromatogram.

### 3.9. Statistical Analysis

Data collected on various growth, yield, and quality parameters during two years of the study were checked for normality before conducting analysis of variance and later subjected to combined analysis of variance (ANOVA) using the mixed model (proc GLIMMMIX, SAS v 9.3. SAS Institute, Inc., Cary, NC, USA). Statistical significance among irrigation levels and BRs over two years was estimated by a pooled analysis of variance as applicable to split-plot design [86]. The significant difference was tested using Tukey’s least significance difference (LSD) test (α = 0.05).

## 4. Conclusions

Severe deficit irrigation (25% CPE) drastically reduced the chia seed yield to as high as 50%, whereas bioregulators are effective in reducing this loss. Our study reveals that even with a 25% reduction in applied water, it is possible to retain as much seed yield as I_100_ if potassium nitrate, thiourea, or salicylic acid are applied to chia. Maximum water productivity could be achieved, with a 50% reduction in applied water (50% CPE) at the cost of a 10% seed yield penalty compared to I_100_ without BRs, suggesting that this crop can be remunerative even under water-scarce regions. Oil and omega–3 content of chia remain unchanged even after a reduction in water supply by 40% when potassium nitrate and thiourea are sprayed. Therefore, the use of bioregulators can save water to the tune of almost 40% without loss of seed yield, oil content, or omega–3 content. Therefore, results evidenced the advantage of BRs such as potassium nitrate, thiourea, and salicylic acid to schedule deficit irrigation in chia under semiarid agroclimates of India.

## Figures and Tables

**Table 1:** Meteorological data of the experimental site during cropping season (2020–21 and 2021–22).

Month	T_max_ (°C)		T_min_ (°C)		Mean Relative Humidity (%)		Rainfall (mm)		Cumulative Pan Evaporation (mm)	
	2020–21	2021–22	2020–21	2021–22	2020–21	2021–22	2020–21	2021–22	2020–21	2021–22
November	30.9	30.4	17.4	18.8	63.0	68.2	0.0	14.6	141.3	123.7
December	29.8	28.2	14.1	14.8	62.5	71.3	0.0	5.4	128.6	83.8
January	30.7	27.8	16.7	12.8	64.9	66.1	10.6	0.0	125.3	112.5
February	31.4	31.9	14.0	13.5	52.0	55.0	0.0	0.0	147.9	155.8
Avg./total *	30.7	29.5	15.5	14.9	60.6	65.1	10.6 *	20 *	543.1 *	475.8 *

* total values.

**Table 2:** Seed, oil, and omega–3 yield of chia as influenced by deficit irrigation regimes and bioregulators.

I/BRs	Seed Yield (kg ha^–1^)					Oil Yield (kg ha^–1^)					Omega–3 Yield (kg ha^–1^)				
	I_25_	I_50_	I_75_	I_100_	Mean BRs	I_25_	I_50_	I_75_	I_100_	Mean BRs	I_25_	I_50_	I_75_	I_100_	Mean BRs
No BRs	300.1 n	538.8 j	666.0 f	687.3 de	548.0e	92.6 m	169.9 h	215.3 e	224.9 d	171.7 e	47.4 q	87.4 l	112.2 h	118.6 fg	91.4 f
KO	308.7 mn	550.1 ij	673.6 ef	700.2 d	558.2d	95.6 lm	173.3 h	217.2 e	229.1 d	178.8 d	49.1 pq	89.7 l	113.5 h	121.0 f	93.3 e
SA	331.1 kl	604.4 g	718.6 c	737.7 ab	597.9 b	104.8 i–k	193.4 f	235.3 a–c	243.1 ab	194.2 b	54.3 m–o	101.5 j	124.5 e	131.0 bc	102.8 c
PS	318.9 lm	560.1 hi	687.7 de	728.9 a–c	573.9 c	100.1 kl	178.9 g	223.7 d	240.5 a–c	185.8 c	51.4 no	93.0 k	117.3 g	127.8 d	97.4 d
SB	323.8 lm	567.9 h	688.7 de	731.4 a–c	577.9 c	101.8 jk	181.8 g	224.9 d	241.1 ab	187.4 c	52.6 op	95.0 k	118.6 fg	127.8 d	98.5 d
TU	334.1 kl	608.3 g	724.6 bc	738.3 ab	601.3 b	106.2 ij	196.0 f	238.3 bc	244.2 a	196.2 b	55.4 mn	103.7 ij	127.2 de	133.8 ab	105.0 b
KN	344.8 k	615.0 g	738.5 a	742.1 a	610.1 a	109.1 i	198.5 f	242.2 ab	245.0 a	198.7 a	57.0 m	104.8 i	129.7 cd	134.39 a	106.5 a
Mean I	323.1 d	577.8 c	699.7 b	723.7 a		101.5 d	184.5 c	228.2 b	238.3 a		52.51 d	96.49 c	120.47 b	127.82 a	
Pooled analysis (Year × Irrigation × Bioregulators)															
*p* value	Y	<0.0001	Y × I	<0.0001		Y	<0.0001	Y × I	<0.0001		Y	<0.0001	Y × I	<0.0001	
	I	<0.0001	Y × BRs	0.0002		I	<0.0001	Y × BRs	<0.0001		I	<0.0001	Y × BRs	<0.0001	
	BRs	<0.0001	Y × I × BRs			BRs	<0.0001	Y × I × BRs	0.0003		BRs	<0.0001	Y × I × BRs	0.0023	

KO, SA, PS, SB, TU, KN, and no BR denote kaolin, salicylic acid, potassium silicate, sodium benzoate, thiourea, potassium nitrate, and control, respectively. Y, BRs, I denote year, bioregulators, and irrigation, respectively. Means followed by the same letter(s) within a column are not significantly different.

**Table 3:** Changes in yield attributes of chia under deficit irrigation regimes and bioregulators (BRs).

	Number of Spikes per Plant	Spike Length (cm)	1000-Seed Weight (g)	Oil Content of Seeds (%)	Harvest Index (%)
Irrigation					
I_25_	18.67 d	13.98 d	1.13 d	31.39 d	12.35 d
I_50_	21.03 c	16.12 c	1.16 c	31.91 c	19.03 b
I_75_	23.49 b	19.01 b	1.20 b	32.60 b	20.20 a
I_100_	24.76 a	20.17 a	1.23 a	32.91 a	17.21 c
*p* value	<0.0001	<0.0001	<0.0001	<0.0001	<0.0001
Bioregulators					
No BRs	20.19 d	15.07 c	1.17 c	31.85 d	17.81 a
KO	20.22 d	14.62 c	1.18 a–c	31.86 d	17.97 a
SA	22.72 b	19.42 a	1.19 a	32.33 a–c	17.16 b
PS	21.48 c	16.48 b	1.18 a–c	32.20 c	17.20 b
SB	21.37 c	16.57 b	1.18 bc	32.26 bc	17.22 b
TU	23.63 a	19.39 a	1.19 ab	32.49 a	16.49 c
KN	24.28 a	19.68 a	1.19 a	32.43 ab	16.54 c
*p* value	<0.0001	<0.0001	<0.0001	<0.0001	<0.0001
Year					
2020–21	21.9 a	17.11 b	1.19 a	31.77 b	16.91 b
2021–22	22.0 a	17.52 a	1.17 b	32.64 a	17.49 a
*p* value	0.810	0.035	<0.0001	<0.0001	<0.0001

KO, SA, PS, SB, TU, KN, and no PBR denote kaolin, salicylic acid, potassium silicate, sodium benzoate, thiourea, potassium nitrate, and control, respectively. Y, B, I denote year, bioregulators, and irrigation, respectively. I_25_, I_50_, I_75_, and I_100_ denote irrigation regimes at 25, 50, 75, and 100% CPE, respectively. Means followed by the same letter(s) within a column are not significantly different.

**Table 4:** Changes in fatty acid composition of chia seed oil affected by deficit irrigation regimes and bioregulators (BRs).

Treatments	Palmitic Acid (%)	Stearic Acid (%)	Oleic Acid (%)	Linoleic Acid (%)	Linolenic Acid (%)	Saturated Fatty Acids (%)	Polyunsaturated Fatty Acids (%)
Irrigation							
I_25_	9.92 a	6.24 a	9.16 a	22.90 d	51.72 d	16.16 a	74.63 d
I_50_	9.80 a	5.65 b	8.88 b	23.39 c	52.24 c	15.44 b	75.64 c
I_75_	9.57 b	5.01 c	8.63 c	23.98 b	52.77 b	14.59 c	76.75 b
I_100_	9.00 c	4.70 d	8.29 d	24.36 a	53.62 a	13.70 d	77.98 a
*p* value	<0.0001	<0.0001	<0.0001	<0.0001	<0.0001	<0.0001	<0.0001
Bioregulators							
No BRs	10.00 a	5.76 a	9.11 a	23.21 f	51.89 f	15.76 a	75.11 f
KO	9.84 ab	5.86 a	8.85 b	23.33 e	52.06 e	15.71 a	75.39 e
SA	9.36 d	5.28 c	8.54 d	23.97 a	52.80 b	14.08 d	76.77 c
PS	9.64 c	5.47 b	8.91 b	23.71 c	52.25 d	15.11 c	75.96 d
SB	9.75 bc	5.52 b	8.69 c	23.56 d	52.43 c	14.64 b	76.00 d
TU	9.17 d	5.06 d	8.55 d	23.83 b	53.30 a	15.28 e	77.14 b
KN	9.22 d	4.86 e	8.52 d	24.01 a	53.37 a	14.24 f	77.38 a
*p* value	<0.0001	<0.0001	<0.0001	<0.0001	<0.0001	<0.0001	<0.0001
Year							
2020–21	9.55 a	5.39 a	8.73 b	23.62 b	52.50 b	14.95 b	76.12 b
2021–22	9.59 a	5.41 a	8.76 a	23.70 a	52.67 a	15.00 a	76.38 a
*p* value	0.364	0.382	0.012	<0.0001	<0.0001	0.010	<0.0001

KO, SA, PS, SB, TU, KN, and no PBR denote kaolin, salicylic acid, potassium silicate, sodium benzoate, thiourea, potassium nitrate, and control, respectively. Y, B, I denote year, bioregulators, and irrigation, respectively. I25, I50, I75, and I100 denote irrigation regimes at 25, 50, 75, and 100% CPE, respectively. Means followed by the same letter(s) within a column are not significantly different.

**Table 5:** Total amount of water supplied during crop-growing period (2020–21 and 2021–22).

Treatments	Irrigation Water Applied (mm)		Common Irrigation (mm)	Rainfall (mm)		Total Water Supplied (mm)	
	2020–21	2021–22	2020–21 & 2021–22	2020–21	2021–22	2020–21	2021–22
I_25_ (25% CPE)	72.6	66.3	50	10.6	20.0	133.2	136.3
I_50_ (50% CPE)	145.2	132.7	50	10.6	20.0	205.8	202.7
I_75_ (75% CPE)	217.8	199.0	50	10.6	20.0	278.5	269.0
I_100_ (100% CPE)	290.5	265.5	50	10.6	20.0	351.1	335.5
Number of irrigations (scheduled time; DAS)	8 (5, 13, 24, 33, 41, 54, 64, 76)	8 (8, 18, 24, 35, 45, 58, 65, 74)					

## Data Availability

All data are available on in this publication.

## Supplementary Materials

The following supporting information can be downloaded at: https://www.mdpi.com/article/10.3390/plants12030662/s1, Table S1: Interaction effect of irrigation regimes and bio–regulators on yield attributes of chia; Table S2: Interaction effect of irrigation regimes and bio–regulators on fatty acid composition of chia oil.

## References

[1] Silva B.P., Anunciaçao P.C., Matyelka J.C.D.S., Della Lucia C.M., Martino H.S.D., Pinheiro-Sant’Ana H.M. (2017). Chemical composition of Brazilian chia seeds grown in different places. Food Chem..

[2] Migliavacca R.A., Silva T.R.B., Vasconcelos A.L.S., Mourão Filho W., Baptistella J.L.C. (2014). Chia’s cultivation in Brazil: Future and perspectives. J. Agron. Crop Sci..

[3] Felisberto M.H.F., Galvão M.T.E.L., Picone C.S.F., Cunha R.L., Pollonio M.A.R. (2015). Effect of prebiotic ingredients on the rheological properties and microstructure of reduced-sodium and low-fat meat emulsions. LWT Food Sci. Technol..

[4] Pathak P.O., Nagarsenker M.S., Barhate C.R., Padhye S.G., Dhawan V.V., Bhattacharyya D., Viswanathan C.L., Steiniger F., Fahr A. (2015). Cholesterol anchored arabinogalactan for asialoglycoprotein receptor targeting: Synthesis, characterization, and proof of concept of hepatospecific delivery. Carbohydr. Res..

[5] Dick M., Costa T.M., Gomaa A., Subirade M., Rios-Ade O., Flôres S.H. (2015). Edible film production from chia seed mucilage: Effect of glycerol concentration on its physicochemical and mechanical properties. Carbohydr. Polym..

[6] Mansuri S., Kesharwani P., Jain K., Tekade R.K., Jain N.K. (2016). Mucoadhesion: A promising approach in drug delivery system. React. Funct. Polym..

[7] Ayerza H.R., Coates W. (2011). Protein content, oil content and fatty acid profiles as potential criteria to determine the origin of commercially grown chia (*Salvia hispanica* L.). Ind. Crops Prod..

[8] Ayerza R. (2013). Seed composition of two chia (*Salvia hispanica* L.) genotypes which differ in seed colour. Emir. J. Food Agric..

[9] Fernandes S.S., Romani V.P., Filipini G.S., Martins V.G. (2020). Chia seeds to develop new biodegradable polymers for food packaging: Properties and biodegradability. Polym. Eng. Sci..

[10] Zhang F.Y., Wu P.T., Zhao X.N., Cheng X.F. (2012). Water—saving mechanisms of intercropping system in improving cropland water use efficiency. Chin. J. Appl. Ecol..

[11] Kadasiddappa M.M., Rao V.P., Reddy K.Y., Ramulu V., Devi M.U., Reddy S.N. (2017). Effect of Irrigation (Drip/Surface) on Sunflower Growth, Seed and Oil Yield, Nutrient Uptake and Water Use Efficiency—A Review. Agric. Rev..

[12] Acevedo M., Pixley K., Zinyengere N., Meng S., Tufan H., Cichy K., Bizikova L., Isaacs K., Ghezzi–Kopel K., Porciello J. (2020). A scoping review of adoption of climate–resilient crops by small-scale producers in low and middle–income countries. Nat. Plants.

[13] Herman S., Marco G., Cecilia B., Alfonso V., Luis M., Cristián V., Sebastián P., Sebastián A. (2016). Effect of water availability on growth, water use efficiency and omega3 (ALA) content in two phenotypes of chia (*Salvia hispanica* L.) established in the arid Mediterranean zone of Chile. Agric. Water Manag..

[14] Geerts S., Raes D. (2009). Deficit irrigation as an on-farm strategy to maximize crop water productivity in dry areas. Agric. Water Manag..

[15] De Falco B., Fiore A., Bochicchio R., Amato M., Lanzotti V. (2018). Metabolomic analysis by UAE-GC MS and antioxidant activity of *Salvia hispanica* (L.) seeds grown under different irrigation regimes. Ind. Crops Prod..

[16] Ozer H., Cobana F., Sahinb U., Orsb S. (2020). Response of black cumin (*Nigella sativa* L.) to deficit irrigation in a semi–arid region: Growth, yield, quality, and water productivity. Ind. Crops Prod..

[17] Wakchaure G.C., Minhas P.S., Ratnakumar P., Choudhary R.L. (2016). Optimising supplemental irrigation for wheat (*Triticum aestivum L*.) and the impact of plant bio–regulators in a semi–arid region of Deccan Plateau in India. Agric. Water Manag..

[18] Harisha C.B., Asangi H.A., Singh R. (2019). Growth, yield, water use efficiency of coriander (*Coriandrum sativum*) affected by irrigation levels and fertigation. Indian J. Agric. Sci..

[19] Hassanein R.A., Amin A.A.E., Rashad E.M., Ali A. (2015). Effect of thiourea andsalicylic acid on antioxidant defense of wheat plants under drought stress. Int. J. Chem. Tech. Res..

[20] Wakchaure G.C., Minhas P.S., Meena K.K., Kumar S., Rane J. (2020). Effect of plant growth regulators and deficit irrigation on canopy traits, yield, water productivity and fruit quality of eggplant (*Solanum melongena* L.) grown in the water scarce environment. J. Environ. Manag..

[21] Ratnakumar P., Khan M.I.R., Minhas P.S., Farooq M.A., Sultana R., Per T.S., Deokate P.P., Khan N.A., Singh Y., Rane J. (2016). Can plant bio-regulators minimize crop productivity losses caused by drought, salinity and heat stress? An integrated review. J. Appl. Bot. Food Qual..

[22] Kaya C., Ashraf M., Sönmez O. (2015). Promotive effect of exogenously applied thiourea on key physiological parameters and oxidative defense mechanism in salt–stressed *Zea mays* L. plants. Turk. J. Bot..

[23] Srivastava A.K., Ratnakumar P., Minhas P.S., Suprasanna P. (2016). Plant bio–regulators for sustainable agriculture; integrating redox signaling as a possible unifying mechanism. Adv. Agron..

[24] Bhunia S.R., Verma I.M., Sahu M.P., Sharma N.C., Balai K. (2015). Effect of drip irrigation and bioregulators on yield, economics and water use of fenugreek (*Trigonella foenum-graecum*). J. Spices Aromat. Crops..

[25] Gimeno V., Díaz–López L., Simón–Grao S., Martínez V., Martínez–Nicolás J.J., García–Sánchez F. (2014). Foliar potassium nitrate application improves the tolerance of *Citrus macrophylla* L. seedlings to drought conditions. Plant Physiol. Biochem..

[26] Shahi S., Kumar R., Srivastava M. (2019). Response of black gram (*Vigna mungo*) to potassium under water stress condition. Ann. Plant Soil Res..

[27] Aslam A., Khan S., Ibrar D., Irshad S., Bakhsh A., Gardezi S.T.R., Ali M., Hasnain Z., Al–Hashimi A., Noor M.A. (2021). Defensive impact of foliar applied potassium nitrate on growth linked with improved physiological and antioxidative activities in sunflower (*Helianthus annuus* L.) hybrids grown under salinity stress. Agronomy.

[28] Shakirova F.M., Sakhabutdinova A.R., Bezrukova M.V., Fathudinova R.A., Fathudinova D.R. (2003). Changes in hormonal status of wheat seedlings induced by salicylic acid and salinity. Plant Sci..

[29] Askari E., Ehsanzadeh P. (2015). Drought stress mitigation by foliar application of salicylic acid and their interactive effects on physiological characteristics of fennel (*Foeniculum vulgare* Mill.) genotypes. Acta Physiol. Plant.

[30] Hesami S., Nabizadeh E., Rahimi A., Rokhzadi A. (2012). Effects of salicylic acid levels and irrigation intervals on growth and yield of coriander (*Coriandrum sativum*) in field conditions. Environ. Exp. Biol..

[31] Farjam S., Siosemardeh A., Kazemi–Arbat H., Yarnia M., Rokhzadi A. (2014). Response of chickpea (*Cicer arietinum* L.) to exogenous salicylic acid and ascorbic acid under vegetative and reproductive drought stress condition. J. Appl. Bot. Food Qual..

[32] Abdallah M.M.S., El–Bassiouny H.M.S., AbouSeeda M.A. (2019). Potential role of kaolin or potassium sulfate as anti-transpirant on improving physiological, biochemical aspects and yield of wheat plants under different watering regimes. Bull. Natl. Res. Cent..

[33] Mahmoudian M., Rahemi M., Karimi S., Yazdani N., Tajdini Z., Sarikhani S., Vahdati K. (2021). Role of kaolin on drought tolerance and nut quality of Persian walnut. J. Saudi Soc. Agric. Sci..

[34] Faghih S., Zamani Z., Fatahi R., Liaghat A. (2019). Effects of deficit irrigation and kaolin application on vegetative growth and fruit traits of two early ripening apple cultivars. Biol. Res..

[35] Wang M., Wang R., Mur L.A.J., Ruan J., Shen Q., Guo S. (2021). Functions of silicon in plant drought stress responses. Hortic. Res..

[36] Gong H., Zhu X., Chen K., Wang S., Zhang C. (2005). Silicon alleviates oxidative damage of wheat plants in pots under drought. Plant Sci..

[37] Gunes A., Pilbeam D.J., Inal A., Coban S. (2008). Influence of silicon on sunflower cultivars under drought stress, I: Growth, antioxidant mechanisms, and lipid peroxidation. Commun. Soil Sci. Plant Anal..

[38] Shen X., Zhou Y., Duan L., Li Z., Eneji A.E., Li J. (2010). Silicon effects on photosynthesis and antioxidant parameters of soybean seedlings under drought and ultraviolet–B radiation. J. Plant Physiol..

[39] Helaly M.N., El–Hoseiny H., El–Sheery N.I., Rastogi A., Kalaji H.M. (2017). Regulation and physiological role of silicon in alleviating drought stress of mango. Plant Physiol. Biochem..

[40] Nawaz M.A., Huang Y., Bie Z., Reiter R.J., Niu M., Hameed S. (2016). Melatonin: Current status and future perspectives in plant science. Front. Plant Sci..

[41] Wakchaure G.C., Minhas P.S., Meena K.K., Singh N.P., Hegade P.M., Sorty A.M. (2018). Growth, bulb yield, water productivity and quality of onion (*Allium cepa* L.) as affected by deficit irrigation regimes and exogenous application of plant bio–regulators. Agric. Water Manag..

[42] Ayerza R. (1995). Oil content and fatty acid composition of chia (*Salvia hispanica* L.) from five north–western locations in Argentina. J. Am. Oil Chem. Soc..

[43] Baginsky C., Arenas J., Escobar H., Garrido M., Valero N., Tello D., Pizarro L., Valenzuela A., Morales L., Silva H. (2016). Growth and yield of chia (*Salvia hispanica* L.) in the Mediterranean and desert climates of Chile. Chilean J. Agric. Res..

[44] Hergert G.W., Margheim J.F., Pavlista A.D., Martin D.L., Isbell T.A., Supalla R.J. (2016). Irrigation response and water productivity of deficit to fully irrigated spring camelina. Agric. Water Manag..

[45] Pavlista A.D., Isbell T.A., Baltensperger D.D., Hergert G.W. (2011). Planting date and development of spring–seeded irrigated canola, brown mustard and camelina. Ind. Crops Prod..

[46] Santos R.F., Bassegio D., Silva M.A. (2017). Yield and production components of safflower genotypes affected by irrigation at phenological stages. Agric. Water Manag..

[47] Pandey M., Srivastava A.K., D’Souza S.F., Penna S. (2013). Thiourea, a ROS scavenger, regulates source to sink relationship to enhance crop yield and oil content in *Brassica juncea* (L.). PLoS ONE.

[48] Neupane D., Juan K.Q., Mclennon E., Davison J., Lawry T. (2020). Camelina production parameters response to different irrigation regimes. Ind. Crop. Prod..

[49] Ghamarnia H., Khosravy H., Sepehri S. (2010). Yield and water use efficiency of (*Nigella sativa* L.) under different irrigation treatments in a semi arid region in the West of Iran. J. Med. Plants Res..

[50] Ebrahimian E., Seyyedi S.M., Bybordi A., Damalas C.A. (2019). Seed yield and oil quality of sunflower, safflower, and sesame under different levels of irrigation water availability. Agric. Water Manag..

[51] Morsy A.R., Mohamed A.M., Abo–Marzoka E.A., Megahed M.A.H. (2018). Effect of Water Deficit on Growth, Yield and Quality of Soybean Seed. J. Plant Prod. Mansoura Univ..

[52] Raza M.A.S., Saleem M.F., Khan I.H. (2018). Amelioration in growth and physiological efficiency of sunflower (*Helianthus annuus* L.) under drought by potassium application. Commun. Soil Sci. Plant Anal..

[53] Vekaria G.B., Talpada M.M., Sutaria G.S., Akbari K.N. (2013). Effect of foliar nutrition of potassium nitrate on the growth and yield of greengram (*Vigna Rabiata* L.). Legum. Res..

[54] Singh A., Singh A.K., Aswin C. (2017). Effect of hydrogel and thiourea on yield, quality and nutrient uptake of Indian mustard under moisture stress condition. Res. Crop..

[55] Waqas M.A., Kaya C., Riaz A., Farooq M., Nawaz I., Wilkes A., Li Y. (2019). Potential mechanisms of abiotic stress tolerance in crop plants induced by thiourea. Front. Plant Sci..

[56] Silva H., Arriagada C., Campos–Saez S., Baginsky C., Castellaro–Galdames G., Morales–Salinas L. (2018). Effect of sowing date and water availability on growth of plants of chia (*Salvia hispanica* L.) established in Chile. PLoS ONE.

[57] Furtado G.F., Xavier D.A., Andrade E.M.G., de Lima G.S., Chaves L.H.G., de Vasconcelos A.C.F., Wanderley J.A.C. (2016). Growth and physiological responses of sunflower grown under levels of water replacement and potassium fertilization. Afr. J. Agric. Res..

[58] Shoukat M.R., Leghari S.J., Ahmad N., Virk A.L., Haider F.U., Rehmani M.I.A., Laraib I. (2022). Effects of foliar applied thiourea on maize physiology, growth and yield (*Zea mays* L.) under shaded conditions. J. Plant Nutr..

[59] Nkrumah P., Amadu A.M., Ayeh K.O. (2021). Influence of salicylic acid and potassium nitrate on plant height and flowering time of groundnut (*Arachis hypogaea* L.) under varying salinity and drought–induced stresses. Ghana J. Sci..

[60] Akladious S.A. (2014). Influence of thiourea application on some physiological and molecular criteria of sunflower (*Helianthus annuus* L.) plants under conditions of heat stress. Protoplasma.

[61] Wakchaure G.C., Minhas P.S., Kumar S., Khapte P.S., Meena K.K., Rane J., Pathak H. (2021). Quantification of water stress impacts on canopy traits, yield, quality and water productivity of onion (*Allium cepa* L.) cultivars in a shallow basaltic soil of water scarce zone. Agric. Water Manag..

[62] Halli H.M., Angadi S., Kumar A., Govindasamy P., Madar R., Baskar V.D.C., Elansary H.O., Tamam N., Abdelbacki A.M., Abdelmohsen S.A. (2021). Assessment of planting method and deficit irrigation impacts on physio-morphology, grain yield and water use efficiency of maize (*Zea Mays* L.) on vertisols of semi-arid tropics. Plants.

[63] Silva G.F.C., Goncalves A.C.A., Da Silva C.A., Nanni M.R., Facco C.U., Cenzar E., Da Silva A.A. (2016). NDVI Responses to water stress in different phenological stages in culture bean. J. Agron..

[64] Taghvaeian S., Comas L., DeJonge K.C., Trout T.J. (2014). Conventional and simplified canopy temperature indices predict water stress in sunflower. Agric. Water Manag..

[65] Ashfaq A., Hussain N., Athar M. (2015). Role of potassium fertilizers in plant growth, crop yield and quality fiber production of cotton. Fuuast. J. Biol..

[66] Flagella Z., Rotunno T., Tarantino E., Di Caterina R., De Caro A. (2002). Changes in seed yield and oil fatty acid composition of high oleic sunflower (*Helianthus annuus* L.) hybrids in relation to the sowing date and water regime. Eur. J. Agron..

[67] Joshan Y., Sani B., Jabbari H., Mozafari H., Moaveni P. (2019). Effect of drought stress on oil content and fatty acids composition of some safflower genotypes. Plant Soil Environ..

[68] Bellaloui N., Hu Y., Mengistu A., Kassem M.A., Abel C.A. (2013). Effects of foliar boron application on seed composition, cell wall boron, and seed δ^15^N and δ^13^C isotopes in water–stressed soybean plants. Front. Plant Sci..

[69] Amirkhiz K.F., Dehaghi M.A., Sanavy S.A.M.M., Rezazadeh A. (2021). Evaluation of changes in fatty acid profile, grain, and oil yield of *Carthamus tinctorius* L. in response to foliar application of polyamine compounds under deficit irrigation conditions. Ind. Crops Prod..

[70] Upchurch R.G. (2008). Fatty acid unsaturation, mobilization, and regulation in the response of plants to stress. Biotechnol. Lett..

[71] Samanci B., Ozkaynak E. (2003). Effect of Planting Date on Seed Yield, Oil Content and Fatty Acid Composition of Safflower (*Carthamus tinctorius*) Cultivars Grown in the Mediterranean Region of Turkey. J. Agron. Crop Sci..

[72] Oliva M.L., Shannon J.G., Sleper D.A., Ellersieck M.R., Cardinal A.J., Paris R.L., Lee J.D. (2006). Stability of fatty acid profile in soybean genotypes with modified seed oil composition. Crop Sci..

[73] Hasanuzzaman M., Bhuyan M.H.M.B., Zulfiqar F., Raza A., Mohsin S.M., Al–Mahmud J., Masayuki Fujita M., Fotopoulos V. (2020). Reactive oxygen species and antioxidant defense in plants under abiotic stress: Revisiting the crucial role of a universal defense regulator. Antioxidants.

[74] Amiri–Darban N., Nourmohammadi G., Rad A.H.S., JavadMirhadi S.M., Heravan I.M. (2020). Potassium sulfate and ammonium sulfate affect quality and quantity of camelina oil grown with different irrigation regimes. Ind. Crops Prod..

[75] Bellaloui N., Smith J.R., Gillen A.M., Ray J.D. (2011). Effects of maturity, genotypic background, and temperature on seed mineral composition in nearisogenic soybean lines in the early soybean production system. Crop Sci..

[76] Tezara W., Mitchell V., Driscoll S.P., Lawlor D.W. (2002). Effects of water deficit and its interaction with CO_2_ supply on the biochemistry and physiology of photosynthesis in sunflower. J. Exp. Bot..

[77] Dhaker R.C., Dubey R.K., Tiwari R.C., Dubey S.K. (2016). Water use, yield and economics of fenugreek (*Trigonella foenum–graecum* L.) under varying IW–CPE ratios and fertilizer levels in South West Rajasthan. Legume Res..

[78] Waraich E.A., Ahmad R., Ashraf M.Y., Saifullah, Ahmad M. (2011). Improving agricultural water use efficiency by nutrient management in crop plants. Acta Agric. Scand. Soil Plant Sci..

[79] Minhas P.S., Bal S.K., Kumar S.P., Singh Y., Wakchaure G.C., Ghadge S.V., Nangare D.D., Taware P.B. (2015). Turning Basaltic Terrain into Model Research Farm: Chronicle Description.

[80] Rajagopal V., Choudhary R.L., Kumar N., Krishnani K.K., Singh Y., Bal S.K., Minhas P.S., Singh N.P. (2018). Soil Health Status of NIASM Southern Farm Land.

[81] Pask A.J.D., Pietragalla J., Mullan D.M., Reynolds M.P. (2012). Physiological Breeding II: A Field Guide to Wheat Phenotyping.

[82] Bandyopadhyay K.K., Pradhan S., Sahoo R.N., Singh R., Gupta V.K. (2014). Characterization of water stress and prediction of yield of wheat using spectral indices under varied water and irrigation management practices. Agric. Water Manag..

[83] Howell T.A., Arntzem C., Ritter E. (1994). Irrigation Engineering, Evapotranspiration.

[84] Firestone D., AOCS (2017). American Oil Chemist’s Society Official Method Ba 3–38. Sampling and analysis of oilseed by–products. Official Methods and Recommended Practices of the AOCS.

[85] Firestone D., AOCS (2017). American Oil Chemists’ Society Official Method Ce 2–66: Preparation of Methyl Esters of Fatty Acids. Official Methods and Recommended Practices of the AOCS.

[86] Gomez K.A., Gomez A.A. (1984). Statistical Procedures for Agricultural Research.

