# Supplementary material for: Promising Bioregulators for Higher Water Productivity and Oil Quality of Chia under Deficit Irrigation in Semiarid Regions
Source: Plants (Basel). 2023 Feb 2;12(3):662. doi: 10.3390/plants12030662 (PMC9921998; doi:10.3390/plants12030662)
Supplement: Supplementary file 1 [file plants-12-00662-s001.zip › plants-2157524-supplementary.pdf]

Table S1

Interaction effect of irrigation regimes and bio–regulators on yield attributes of chia

Treatments	Number of spikes per plant	Spike length (cm)	1000 seed weight (g)	Oil content (%)	Harvest index
Interaction effect of I and BRs					
I_{25}–Control	15.26 l	10.87 h	1.12 k	30.86 l	14.26h
I_{25} –KO	15.10 l	10.16 h	1.13 k	31.00 kl	14.33h
I_{25} –SA	20.72 h–j	17.13 d–f	1.14 g–k	31.65 h–j	11.48jk
I_{25} –PS	17.62 k	12.03 h	1.13 jk	31.38 jk	12.84i
I_{25}–SB	17.66 k	11.99 h	1.13 k	31.44 i–k	12.31ij
I_{25}–TU	21.98 g–i	17.77 c–f	1.14 h–k	31.80 g–h	10.48k
I_{25} –KN	22.34 f–h	17.95 c–e	1.14 i–k	31.62 i–h	10.78 k
I_{50} –Control	18.63 k	12.69 gh	1.16 g–k	31.51 h–j	19.33 b–d
I_{50} –KO	19.05 jk	12.15 h	1.17 e–i	31.53 ij	19.37 b–d
I_{50} –SA	21.84 g–i	18.66 b–e	1.17 e–g	32.00 h–j	19.45 b–d
I_{50} –PS	20.47 ij	15.40 fg	1.16 f–j	32.10 f–i	18.87 cd
I_{50}– SB	20.40 ij	16.32 ef	1.16 g–k	32.00 f–h	19.30 b–d
I_{50}– TU	22.96 d–g	19.28 a–d	1.17 e–h	32.23 e–g	18.37d–f
I_{50}– KN	23.86 b–f	19.47 a–d	1.17 e–i	32.28 d–g	18.50 de
I_{75}– Control	22.80 d–g	17.17 d–f	1.19 d–f	32.32 c–f	20.40 ab
I_{75}– KO	22.46 e–g	16.93 d–f	1.19 d–f	32.24 e–g	20.88 a
I_{75}– SA	23.36 c–g	20.40 a–c	1.21 a–d	32.69 a–d	20.38 ab
I_{75} –PS	23.41 c–g	18.84 a–e	1.19 c–f	32.52 b–e	19.81 a–c
I_{75} –SB	23.10 d–g	18.70 a–e	1.20 b–e	32.65 a–e	20.13 ab
I_{75}–TU	24.30 a–d	20.04 a–c	1.21 a–d	32.89 ab	19.93a–c
I_{75}– KN	25.05 a–c	20.98 ab	1.22 a–d	32.80 a–c	19.88 a–c
I_{100}– Control	24.10 b–e	19.57 a–d	1.22 ab	32.69 a–e	17.25 fg
I_{100}– KO	24.27 a–d	19.22 a–d	1.22 ab	32.70 a–e	17.30 fg
I_{100}– SA	24.98 a–c	21.49 a	1.23 ab	32.95 a–d	17.34 e–g
I_{100}– PS	24.44 a–d	19.63 a–d	1.23 ab	32.98 ab	17.28fg
I_{100}– SB	24.34 a–d	19.14 a–d	1.22 ab	32.96 ab	17.12g
I_{100}–TU	25.28 ab	20.81 ab	1.23 ab	33.07 a	17.18g
I_{100}– KN	25.89 a	21.14 ab	1.24 a	33.01 a	17.01g
P value	<.0001	<.0001	0.891	0.191	<.0001
Year					
2020–21	21.9a	17.11b	1.19a	31.77b	16.91b
2021–22	22.0a	17.52a	1.17b	32.64a	17.49a
P value	0.810	0.035	<.0001	<.0001	<.0001

KO, SA, PS, SB, TU, KN and no PBR denote Kaolin, salicylic acid, Potassium silicate, sodium benzoate, thiourea, potassium nitrate and control, respectively. Y, B, I denotes year, bio regulators and Irrigation respectively. I25, I50, I75 and I100 denotes irrigation regimes at 25, 50, 75 and 100 %

CPE respectively. Means followed by the same letter, (s) within a column are not significantly differed.

Table S2

Interaction effect of irrigation regimes and bio–regulators on fatty acid composition of chia oil

Interaction effect of irrigation regimes and bio–regulators on yield attributes of chia

Treatments/ Fatty acids	Palmitic acid (%)	Stearic acid (%)	Oleic acid (%)	Linoleic acid (%)	Linolenic acid (%)
Interaction effect					
I_{25}–Control	10.21a	6.838 a	9.43 a	22.28 m	51.22 n
I_{25} –KO	10.18ab	6.732 ab	9.28 a	22.38 m	51.41 mn
I_{25} –SA	9.94a–d	5.902 de	9.02 b	23.24 j	51.87 j–l
I_{25} –PS	9.89a–e	6.352 c	9.35 a	22.97 kl	51.42 mn
I_{25}–SB	9.86 a–f	6.478 bc	9.10 b	22.85 l	51.69 lm
I_{25}–TU	9.58 d–i	5.760 d–f	8.96 b	23.34 ij	52.13 h–k
I_{25} –KN	9.77a–g	5.668 d–f	8.98 b	23.27 ij	52.28 hi
I_{50} –Control	10.20a	5.960 d	9.35 a	23.01 kl	51.46 mn
I_{50} –KO	10.12ab	5.892 de	8.97	23.15 jk	51.76 k–m
I_{50} –SA	9.32 g–j	5.780 de	8.77 cd	23.62 h	52.51 f–h
I_{50} –PS	9.73 a–h	5.940 de	8.98 b	23.31 ij	52.01 i–l
I_{50}– SB	9.92 a–d	5.870 de	8.77 c	23.16 jk	52.25 h–j
I_{50}– TU	9.65 d–i	5.192 gh	8.63 c–f	23.58 hi	52.92 e
I_{50}– KN	9.65 d–i	4.922 h–k	8.69 c–e	23.91 g	52.81 e–g
I_{75}– Control	9.95a–d	5.460 fg	8.96 b	23.47 h	52.13 h–k
I_{75}– KO	9.69 b–i	5.640 ef	8.73 cd	23.67 h	52.24 h–j
I_{75}– SA	9.24 h–j	4.842 i–l	8.49 f–h	24.40 bc	52.91 e
I_{75} –PS	9.75 a–g	4.992 h–j	8.76 cd	24.05 fg	52.43 gh
I_{75} –SB	9.60 d–i	5.120 hi	8.60 d–f	23.90 g	52.76 e–g
I_{75}–TU	9.41 d–i	4.582 l–n	8.49 f–h	24.13 d–f	53.37 cd
I_{75}– KN	9.37 f–j	4.483 mn	8.36 hi	24.22 c–f	53.55 bc
I_{100}– Control	9.64 d–i	4.790 j–l	8.70 c–e	24.07 e–g	52.77 e–g
I_{100}– KO	9.40 e–j	5.202 gh	8.42 g–i	24.10 e–g	52.85 ef
I_{100}– SA	8.95 h–j	4.612 l–n	7.91 l	24.62 a	53.91 b
I_{100}– PS	9.22 ij	4.602 l–n	8.55 e–g	24.50 ab	53.14 de
I_{100}– SB	9.65 c–h	4.640 k–n	8.29 ij	24.34 b–d	53.04 de
I_{100}–TU	8.05 k	4.730 j–m	8.13 jk	24.27 c–e	54.79 a
I_{100}– KN	8.10 k	4.380 n	8.04 kl	24.63 a	54.85 a
P value	<.0001	<.0001	<.0001	<.0001	<.0001
Year					
2020–21	9.55a	5.39a	8.73b	23.62b	52.50b
2021–22	9.59a	5.41a	8.76a	23.70a	52.67a
P value	0..364	0.382	0.012	<.0001	<.0001

KO, SA, PS, SB, TU, KN and no PBR denote Kaolin, salicylic acid, Potassium silicate, sodium benzoate, thiourea, potassium nitrate and control, respectively. Y, B, I denotes year, bio regulators and Irrigation respectively. I25, I50, I75 and I100 denotes irrigation regimes at 25, 50, 75 and 100 % CPE respectively. Means followed by the same letter, (s) within a column are not significantly differed.
